# Near-Infrared to Visible Photon Upconversion with Gold Quantum Rods and Aqueous Photo-Driven Polymerization

**DOI:** 10.1021/jacs.5c08826

**Published:** 2025-07-28

**Authors:** Zhongyu Liu, Xiaolei Hu, Lianshun Luo, Guiying He, Abhrojyoti Mazumder, Ece Gunay, Yitong Wang, Elizabeth C. Dickey, Linda A. Peteanu, Krzysztof Matyjaszewski, Rongchao Jin

**Affiliations:** † Department of Chemistry, 6612 Carnegie Mellon University, Pittsburgh, Pennsylvania 15213, United States; ‡ Department of Materials Science and Engineering, Carnegie Mellon University, Pittsburgh, Pennsylvania 15213, United States

## Abstract

Converting near-infrared (NIR) photons into visible light via triplet–triplet annihilation upconversion (TTA-UC) is a promising strategy for advancing energy, biomedical, and materials science. However, the development of efficient NIR sensitizers remains a major challenge. Here, we report an atomically precise gold quantum rod, Au_42_(PET)_32_ (PET = 2-phenylethanethiolate), as a high-performance photosensitizer for NIR-to-visible TTA-UC. Paired with TES-ADT as an annihilator, the system exhibits a 6.7% quantum yield, a 0.5 eV anti-Stokes shift, and a low threshold intensity of 90 mW/cm^2^. To enable aqueous compatibility, the upconversion nanodroplets are encapsulated with a silica shell, yielding Au_42_/TES-ADT@SiO_2_ nanoparticles (NPs) capable of driving efficient photoinduced atom-transfer radical polymerization (photo-ATRP) and forming hydrogels in water. This system offers a versatile platform for the next generation photopolymerization with NIR light, solar energy utilization and noninvasive biomedical applications.

## Graphical abstract

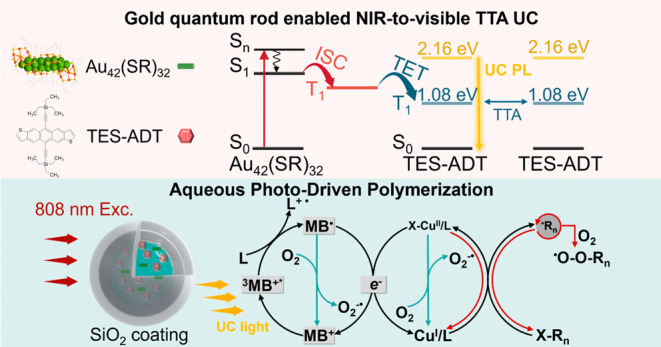

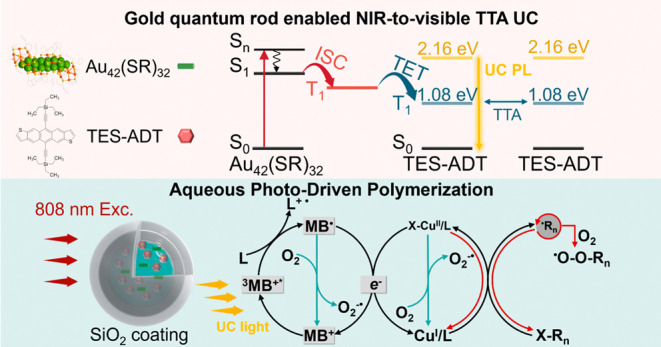

## Introduction

Manipulating light at the quantum level by converting low-energy photons to higher-energy ones drives groundbreaking advancements across various scientific domains, including photocatalysis,^1–4^ photovoltaics^5–8^ and bioapplications.^9–13^ Among the different upconversion mechanisms, triplet–triplet annihilation upconversion (TTA-UC), also known as triplet fusion upconversion, stands out for its high efficiency and versatility.^14–18^ Particularly, the TTA-UC of near-infrared (NIR) light into visible light^19^ is of paramount importance, as NIR photons—which experience minimal scattering^20^—can penetrate deeply into turbid media such as nanoparticle suspensions and biological tissues, whereas visible photons, endowed with higher energy, can drive the photovoltaic effect, initiate photochemical reactions, and induce conformational changes in biomolecules. Consequently, NIR to visible TTA-UC holds promise for enhancing solar cell efficiency,^21^ improving deep-tissue biosensing, and advancing in vivo neuromodulation techniques.^22^

Nevertheless, the development of suitable materials for NIR-to-visible TTA-UC, such as the design of low-energy-loss sensitizers, remains challenging, as each class of sensitizers presents its own limitations.^22^ Organic NIR sensitizers often suffer from inefficient intersystem crossing, leading to low upconversion efficiencies.^23^ Organometallic complexes, though with improved triplet-state generation, typically exhibit low NIR absorption cross sections and are prone to photodegradation, thus, compromising the long-term stability.^4,24^ Lead halide perovskites and other semiconductor colloidal nanocrystals, despite their strong light-harvesting capabilities and tunable optical properties, often incorporate toxic elements, raising concerns over sustainability and biocompatibility and restricting their potential for in vivo applications.^25^

In recent research, gold quantum rods^26^ (QRs) have emerged as a unique and promising alternative of sensitizers. These atomically precise materials consist of tens to hundreds of gold atoms per core and are stabilized by thiolate ligands, and one such system pertains to a series of constant three-atom diameter (0.3 nm) and variable aspect ratios from 6.3 to 18.7 (or 2 to 6 nm length).^26–28^ Their extremely narrow diameter and high aspect ratios induce strong quantum confinement effects, yielding discrete energy levels, tunable peak wavelength, and prolonged exciton lifetime. Moreover, these traits promote strongly polarized excitonic transitions along the longitudinal axis, resulting in intense, sharp absorption peaks between 800 and 2000 nm with absorption coefficients on the order of 10^5^ to 10^6^ M^–1^ cm^–1^.^26^ Importantly, the absence of toxic constituents imparts excellent biocompatibility, further enhancing their potential for future biomedical applications.^29^

A compelling application of NIR-to-visible TTA-UC lies in photocatalysis, such as photoinduced atom-transfer radical polymerization (photo-ATRP). ATRP is one of the most important reversible-deactivation radical polymerization systems that have revolutionized polymer chemistry over the past decades.^30–33^ Particularly, photo-ATRP enables the preparation of well-defined polymers with precise molecular weights, diverse architectures, and high end-group functionality, under precise spatiotemporal control and environmentally benign conditions.^34^ However, current photo-ATRP systems predominantly operate in the 300–680 nm light range, limiting their applicability in biologically relevant and highly scattering environments.^35,36^ Extending photo-ATRP to the NIR region (>800 nm) is of growing interest, as NIR light offers deeper penetration, reduced scattering, enhanced biocompatibility, and minimal photodamage.^37,38^ Despite these advantages, efficient NIR-driven photo-ATRP remains largely unexplored, underscoring the need for innovative photosensitizers capable of harnessing low-energy photons for controlled polymerization.

In this work, we employ an Au_42_(PET)_32_ quantum rod (PET = 2-phenylethanethiolate, hereafter referred to as Au_42_) as a photosensitizer to achieve NIR-to-visible TTA-UC. After screening various annihilators, TES-ADT (5,11-bis­(triethylsilylethynyl)­anthradithiophene) is identified as the optimal one for our TTA-UC system. The interaction between the thiophene groups in TES-ADT and the gold atoms in Au_42_ enhances energy transfer efficiency between the quantum rod and the annihilator/emitter. The Au_42_/TES-ADT system exhibit an upconversion quantum yield of 6.7% (normalized to 100%), achieving a 0.5 eV anti-Stokes shift at a low threshold power density of 90 mW/cm^2^. Moreover, the system demonstrates excellent photostability, and its emitted light successfully initiates a highly efficient and precisely controlled photo-ATRP reaction. To extend its applicability, we further encapsulate the upconversion nanodroplets with a SiO_2_ shell, yielding Au_42_/TES-ADT@SiO_2_ TTA-UC nanoparticles (NPs), which efficiently drive an aqueous photo-ATRP reaction, leading to the formation of a hydrogel. This Au_42_-based system offers a noninvasive and precisely controllable approach to polymerization-based treatments, including localized drug delivery, regenerative medicine, and the development of self-healing materials for electronic and biomedical devices.

## Results and Discussion

### Design of Au_42_ QR TTA-UC System

The Au_42_ QR was synthesized using a method of N-heterocyclic carbene-mediated kinetic control developed by our group.^28^ Single-crystal X-ray diffraction analysis resolved its structure, which exhibited a rod-shaped Au_20_ kernel with six Au­(PET)_2_ motifs anchored along its body and two pairs of interlocked Au_4_(PET)_5_ motifs protecting the two ends (Figure 1a).^27^ This anisotropic architecture gives rise to distinct, chlorophyll-like optical absorption. As shown in Figure 1b (green line), the optical absorption spectrum of Au_42_ exhibits a prominent peak at 806 nm, with an absorption coefficient of ε_806 nm_ = 1.08 × 10^5^ M^–1^ cm^–1^, which is ten times higher than that of Au_25_(SR)_18_,^26^ a commonly used gold nanocluster for NIR light photosensitization. The strong absorption at 806 nm corresponds to the HOMO-to-LUMO transition, with the transition dipole highly polarized along the longitudinal direction. Upon excitation at 806 nm, Au_42_ exhibits dual emission (fluorescence (FL) and phosphorescence (PH)) at 875 and 1045 nm (Figure 1b, shaded area), respectively, with a total photoluminescence quantum yield (PLQY) of 18% in toluene. Deconvolution of the emission spectrum reveals the FL and PH quantum yields to be 10 and 8%, respectively. Time-resolved photoluminescence (PL) analysis revealed the lifetime of triplet state is ∼2.4 μs (Figure S1), which is sufficiently long to enable efficient triplet–triplet energy transfer between Au_42_ and annihilator molecules. Additionally, Au_42_ exhibits very weak absorption between 450 and 700 nm, which is advantageous as this minimizes the reabsorption loss of upconverted photons (visible light, which is within this wavelength range).

**Figure 1:**
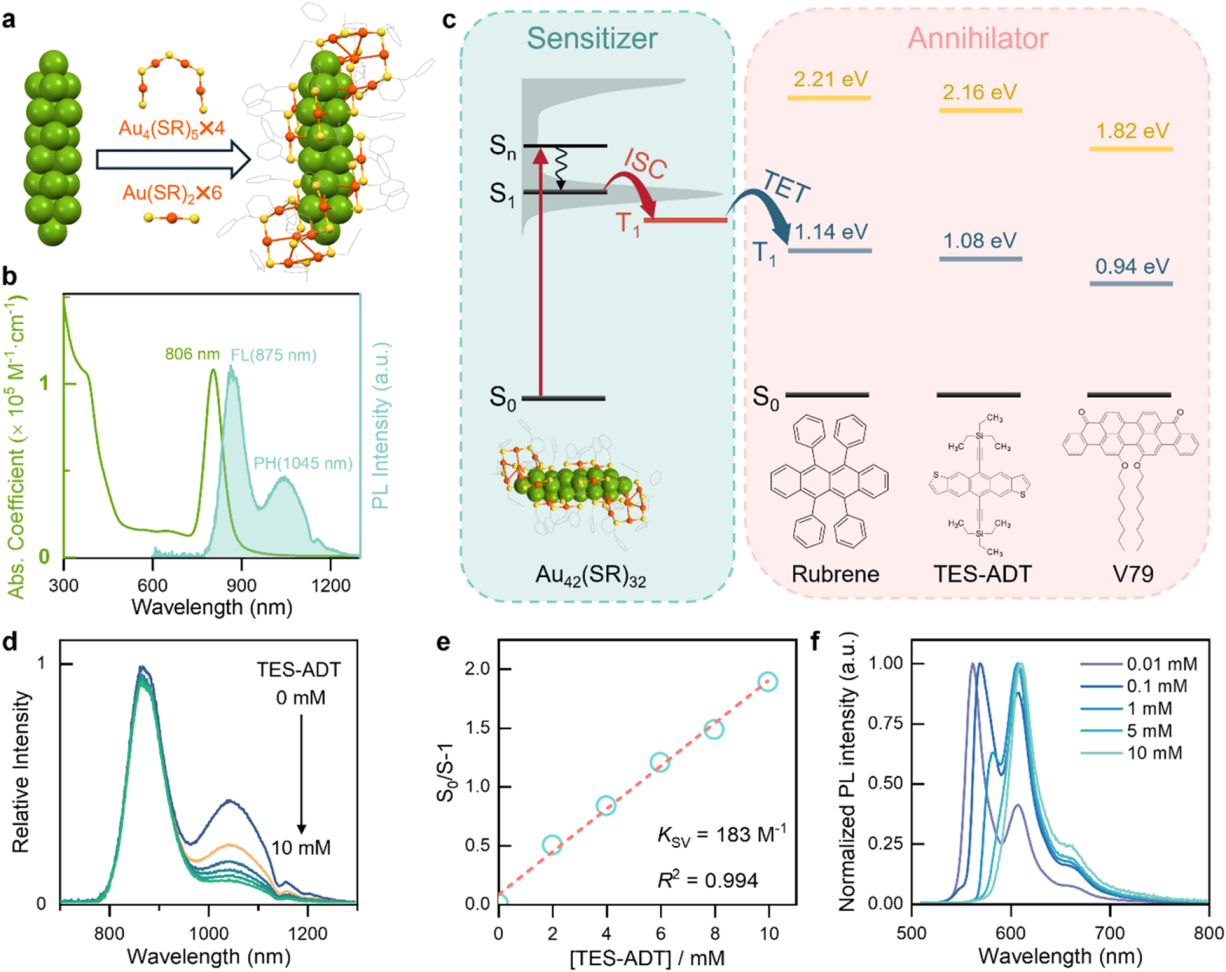
(a) Atomic structure of Au_42_(PET)_32_. Color code: yellow = S, other colors = Au, carbon tails are omitted for clarity. (b) Optical absorption (green line) and photoluminescence (PL, shaded) spectra of Au_42_ dissolved in toluene. (c) Energy diagram illustrating the combinations of Au_42_ as a sensitizer with three annihilators/emitters (V79 = violanthrone 79). (d) Steady-state PL spectra of Au_42_ in deaerated toluene at varying TES-ADT concentrations under 808 nm excitation. (e) Stern–Volmer plot of Au_42_ PL quenching by TES-ADT. (f) Steady-state PL spectra of TES-ADT at different concentrations under 500 nm excitation.

To achieve efficient TTA-UC, effective triplet–triplet energy transfer (TET, Figure 1c) between Au_42_ and the annihilator is essential. Since TET occurs via a Dexter-type energy transfer mechanism^39^ that involves electron exchange, the triplet energy level of the annihilator must be lower than that of the photosensitizer. Based on the phosphorescence emission peak at 1045 nm, the triplet energy level of Au_42_ is estimated to be 1.18 eV. Given this low triplet energy, the selection of suitable annihilators is quite limited, as few molecules possess a matched energy level of triplet for effective energy transfer. Here, we selected three annihilators with matched triplet energy levels—rubrene (1.14 eV), TES-ADT (1.08 eV), and V79 (0.94 eV)—and investigated their TET efficiency with Au_42_ (Figure 1c). The UV–vis absorption and PL emission spectra of the three dyes are shown (see Figure S2), where rubrene and TES-ADT predominantly emit in the yellow region, while V79 primarily emits in the red region.

The TET efficiency was evaluated by measuring the dependence of Au_42_ phosphorescence intensity on the annihilator/emitter concentration, and a Stern–Volmer plot was generated to extract the Stern–Volmer constant (*K*_SV_), providing a quantitative evaluation of the TET efficiency. As shown in Figures 1d and S3, the phosphorescence intensity of Au_42_ is dramatically quenched upon introducing dye molecules into the solution, while the fluorescence intensity remains essentially unchanged. The *K*_SV_ for TES-ADT was determined to be 183 M^–1^ (Figure 1e), while that of rubrene was lower, i.e., 135 M^–1^ (Figure S3b). For V79, the *K*_SV_ was 172 M^–1^ (Figure S3d), slightly lower than TES-ADT. The higher *K*_SV_ of TES-ADT is likely owing to the thiophene group, which interacts with gold atoms,^40^ shortening the distance between the dye and the QR, increasing the collision probability, and thus enhancing dynamic quenching and triplet energy transfer. Under 808 nm excitation, the theoretical anti-Stokes shift of V79 is only 0.29 eV, significantly smaller than the 0.63 and 0.68 eV shifts for TES-ADT and rubrene, respectively. Additionally, our measurements revealed that rubrene exhibits much lower photostability compared to TES-ADT in the designed TTA-UC system, likely due to the instability of rubrene radicals. Considering the TES-ADT’s higher *K*_SV_ value, larger anti-Stokes shift, and excellent photostability, we choose TES-ADT as the most suitable annihilator/emitter for efficient TET and TTA in the Au_42_ TTA-UC system. The concentration dependent PL emission spectra of TES-ADT were measured and are shown in Figure 1f. As the dye concentration increases, the main emission peak shifts from 560 to 610 nm due to reabsorption of emitted photons by TES-ADT itself.

### Characterization of Au_42_ QR Sensitized NIR-to-Visible Upconversion

The designed TTA-UC system, utilizing Au_42_ as the photosensitizer and TES-ADT as the annihilator/emitter, is schematically illustrated in Figure 2a. Experimentally, we excited a deaerated toluene solution of the Au_42_ QR (3 μM) and TES-ADT (10 mM), using an 808 nm continuous-wave laser, and observed yellowish emission from TES-ADT singlet state (Figure 2b, inset). The PL emission spectrum of the solution (Figure 2b) exhibits a prominent TES-ADT emission peak centered at 610 nm, along with the dual emission profile of Au_42_. Notably, this yellowish emission was absent under aerated conditions or in the absence of the nanocluster sensitizer, confirming its origin from upconverted emission via TTA. The anti-Stokes shift (Δas), calculated as the energy difference between the centroid (610 nm) of TES-ADT emission and the excitation laser, is approximately 0.5 eV. Additionally, Au_42_ QR can be excited by longer-wavelength light, ranging from 808 to 980 nm, potentially increasing the anti-Stokes shift beyond 0.8 eV, albeit at the cost of reduced efficiency.

**Figure 2:**
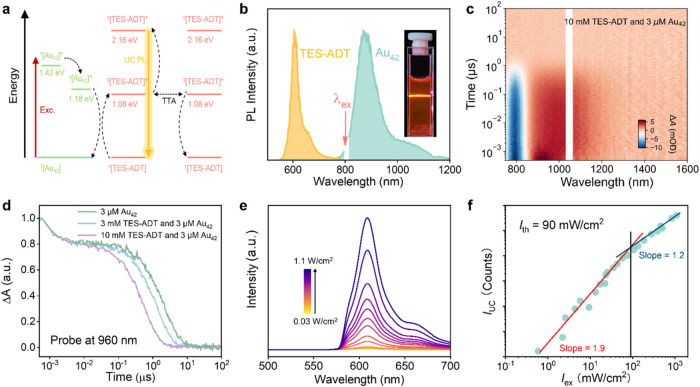
(a) Schematic of the TTA-UC system comprising the Au_42_ sensitizer and TES-ADT annihilator/emitter. (b) TTA-UC emission spectra under 808 nm continuous laser excitation. The inset shows a photograph of the NIR-to-yellow upconversion in a cuvette. (c) Nanosecond transient absorption (TA) spectrum of 3 μM Au_42_ and 10 mM TES-ADT in an N_2_-saturated toluene solution under 808 nm excitation. (d) TA kinetic profiles of Au_42_ and TES-ADT at different concentrations, probed at 960 nm. (e) TTA-UC emission spectra for a 3 μM Au_42_/10 mM TES-ADT toluene solution at various excitation power densities of 808 nm laser. (f) Dependence of the intensity of UC emission on the incident laser fluence for a 3 μM Au_42_/10 mM TES-ADT toluene solution, excited at 808 nm.

The excited-state dynamics of the Au_42_ and TES-ADT system was investigated using nanosecond transient absorption (ns-TA) spectroscopy. Figures 2c,d and S4 show the effect of TES-ADT addition on the TA kinetics and the excited state lifetime of Au_42_. The two-dimensional TA map of Au_42_ (Figure S4a) exhibits a ground-state bleaching (GSB) spanning from 750 to 830 nm and an excited state absorption (ESA) beyond 860 nm. Upon the addition of TES-ADT, these spectral features remain (Figures 2c and S4a). The exciton lifetime of Au_42_ is determined to be 2.4 μs, consistent with the PL lifetime measurements (Figure S1). With increasing TES-ADT concentration, the excited-state lifetime of Au_42_ is shortened (Figure 2d), indicating efficient energy transfer from the clusters to TES-ADT. Specifically, the lifetime of Au_42_ decreases to 1.4 μs at 3 mM TES-ADT and further to 0.61 μs at 10 mM TES-ADT. Stern–Volmer analysis of the extracted excited-state lifetime yields a *K*_SV_ of 298 M^–1^ (Figure S5a), comparable to the value obtained from PL quenching measurements. Because ns-TA directly time-resolves the triplet decay with minimal fluorescence overlap, we consider this K_SV_ determination more reliable. The TET efficiency (Φ_TET_), calculated from the *K*_SV_ values from ns-TA measurements, yields an efficiency of approximately 0.75, indicating that 75% of triplet excitons were successfully transferred from Au_42_ to TES-ADT. This efficiency is comparable to the previously reported values for the PtAg_24_–perylene system.^41^

The observed TTA-UC quantum efficiency (Φ’_UC_; normalized to 100%) was determined to be 6.7(±0.2)% at a TES-ADT concentration of 10 mM using a relative method, with the PL intensity of the Au_42_ fluorescence peak as the reference (Figure 2b). The PL spectra of the Au_42_/TES-ADT UC system were further studied as a function of incident laser fluence (Figure 2e). The integrated intensity of the yellowish upconverted emission from TES-ADT increases with laser fluence. As shown in Figure 2f, the intensity initially exhibits a quadratic dependence (slope = 1.9) on the excitation power before transitioning to a linear dependence (slope = 1.2), with the crossover point at ∼90 mW cm^–2^, defined as the TTA-UC threshold (*I*_th_). Such a quadratic-to-linear dependence is a distinctive hallmark of the TTA-UC process. Below *I*_th_, the concentration of TES-ADT triplet ([^3^TES-ADT*]) is low, and its decay is primarily governed by first-order processes. Consequently, [^3^TES-ADT*] increases linearly with the excitation power, leading to a quadratic growth in TTA-UC intensity. Above *I*_th_, [^3^TES-ADT*] becomes sufficiently high for bimolecular TTA to dominate, resulting in a linear dependence of TTA-UC intensity on the excitation power. It is known that the TTA-UC threshold follows the relation, *I*_th_ = (ε·Φ_TET_·*k*_TTA_·τ_T_^2^)^−1^, where, ε is the absorption coefficient of the sensitizer, Φ_TET_ is the TET quantum yield, *k*_TTA_ is the rate constant of the TTA process, and τ_T_ is the triplet lifetime of the emitter.^41^ The low *I*_th_ observed in this study can be attributed to the strong absorption of Au_42_ at 808 nm and the high Φ_TET_, which are enhanced by the interaction between TES-ADT and Au_42_.

The TTA-UC quantum efficiency can be described by Φ’_UC_ = Φ_ISC_ × Φ_TET_ × Φ_TTA_ × Φ_FL_ × (1 – Φ_ET_) (normalized to 100%),^42^ where, Φ_ISC_ is the intersystem crossing quantum efficiency of Au_42_, Φ_TET_ represents the triplet energy transfer efficiency from Au_42_ to TES-ADT, Φ_TTA_ is the fraction of annihilator triplets that undergo transformation into singlets, Φ_FL_ denotes the photoluminescence quantum yield of TES-ADT, and Φ_ET_ accounts for the emitter triplet quenching by Au_42_. From the quenching experiments, the Φ_TET_ is estimated to be 0.75 as discussed earlier. The Φ_FL_ of TES-ADT at the given concentration is estimated to be 0.38 via a relative method. Φ_TTA_ is an intrinsic property of TES-ADT and has been reported to be approximately 0.30.^43^ Given the extremely low concentration of Au_42_ used in the combination and the high concentration of TES-ADT, the quenching of TES-ADT by Au_42_ can be considered negligible (Figure S5b). Consequently, the Φ_ISC_ of Au_42_ is estimated to be around 0.76.

The Φ’_UC_ of 6.7% represents the highest NIR-to-visible TTA-UC quantum efficiency reported to date compared to other metal nanocluster-based systems and is comparable to many other photosensitizer-based TTA systems (Figure 3, data also summarized in Table S1), including those utilizing organic molecules, metal complexes and semiconductor nanocrystals.^2,6,22,24,25,41,44–51^ Moreover, the Au_42_/TES-ADT system achieves its maximum efficiency at an exceptionally low threshold power density, making it highly energy-efficient. Notably, such a low excitation threshold is rare among systems with high upconversion efficiency (Figure 3). Although this Φ’_UC_ is lower than some previously reported values, it is important to note that the excitation wavelength used here corresponds to the absorption maximum of Au_42_ quantum rods, whereas many other studies were excited at the tail of the absorption peak.^2^ As a result, the overall upconversion brightness in this system may surpass that of other reported cases with higher efficiency. The combination of high upconversion efficiency and low excitation threshold is pivotal in designing a well-balanced system that maximizes the energy efficiency while ensuring practical applicability in real-world scenarios such as bioimaging, optoelectronics, and solar energy conversion.^5^

**Figure 3:**
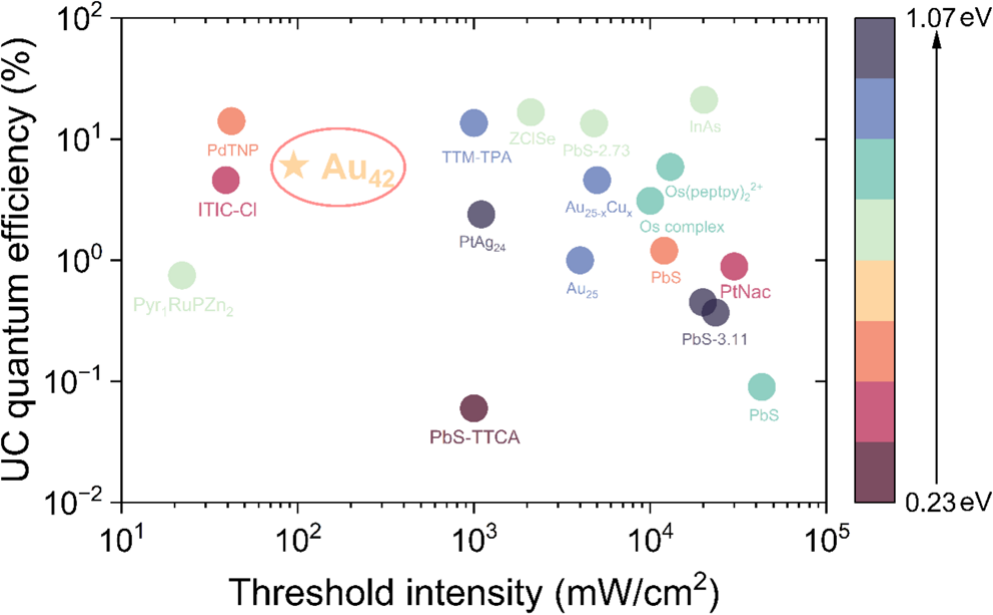
Comparison of NIR-to-visible TTA-UC properties of Au_42_/TES-ADT in this work with other sensitizer mediated upconversion processes. The colors indicate different anti-Stokes shifts, see the color scale bar on the right. (Details summarized in Table S1).

### Stable NIR-to-Visible Upconversion for Photo-ATRP

In addition to the upconversion quantum efficiency, anti-Stokes shift, and threshold intensity, the photostability of the NIR-to-visible TTA-UC system is also crucial for its practical applications. The UC emission intensity under 808 nm photoexcitation at a power density of 1 W/cm^2^ in deaerated toluene is shown in Figure S6, in which the emission intensity of the TTA-UC system decreased by only 14% after 3 h of irradiation, indicating a good photostability. Due to the strong NIR absorption of Au_42_, a photothermal effect can be observed during the test and it is probably the main reason that results in the slight decomposition of Au_42_ and consequently causes the decrease of the TTA intensity. Previously, benzyl mercaptan (BM) protected Au_42_(BM)_32_ was reported to be less stable under the same conditions.^27^ The disparity in stability between BM- and PET-protected NCs has also been observed in other cases and can be attributed to several advantages of PET ligand protection over BM, including (i) stronger Au–S bonding, (ii) lower susceptibility to oxidation, and (iii) more effective surface packing.^52,53^

Given its high UC quantum yield, low threshold intensity and excellent photostability, the Au_42_/TES-ADT TTA-UC system was further explored for its potential in enabling NIR light-induced photo-ATRP reactions. Traditional Cu-catalyzed photo-ATRP was typically performed using UV light irradiation.^34^ However, UV light can have a biocidal effect on biomacromolecules and hinders biorelated applications of photo-ATRP. To address the limitations, recent efforts have focused on developing photosensitizers that can respond to visible or even NIR light. However, the emitted UC light peak centered at 610 nm could not directly excite the ATRP deactivator.^37,38^ Therefore, a cophotocatalyst with strong absorption in the UC emission range as well as capability to drive photo-ATRP is essential. Methylene blue (MB^+^) was reported as an effective photoredox catalyst to enable open-air photo-ATRP at full spectrum range from UV to red light.^37^ Thus, the NIR-light-driven ATRP could be achieved using Au_42_/TES-ADT TTA-UC together with MB^+^ and [X–Cu^II^/TPMA]^+^ deactivator (Figure 4a), where, the emitted UC could subsequently excite MB^+^, leading to the formation of highly reductive MB^•^ radical via a reductive quenching cycle. The as-formed MB^•^ sustains the ATRP process by regenerating the [Cu^I^/TPMA]^+^ activator and, together with [X–Cu^II^/TPMA]^+^, provides control over the polymerization. To test this hypothesis, we carried out the polymerization of oligo­(ethylene oxide) methyl ether methacrylate (OEOMA500, average *M*_n_ = 500) using 2-hydroxyethyl α-bromoisobutyrate (HO-EBiB) as the initiator in a phosphate-buffered saline (PBS) solution containing 1.7% v/v DMSO. The ATRP reaction cocktail (without prior deoxygenation) was sealed in a 0.25 mL reaction tube with a cap, which was then inserted into a glass vial containing the deaerated Au_42_/TES-ADT TTA-UC solution (Figure 4b upper panel). An 808 nm laser with a power density of 1 W/cm^–2^ was used to provide NIR photons (Figure 4b bottom panel). The reaction system can be further optimized to improve the utilization efficiency of incident light, but it is not the focus of this work.

**Figure 4:**
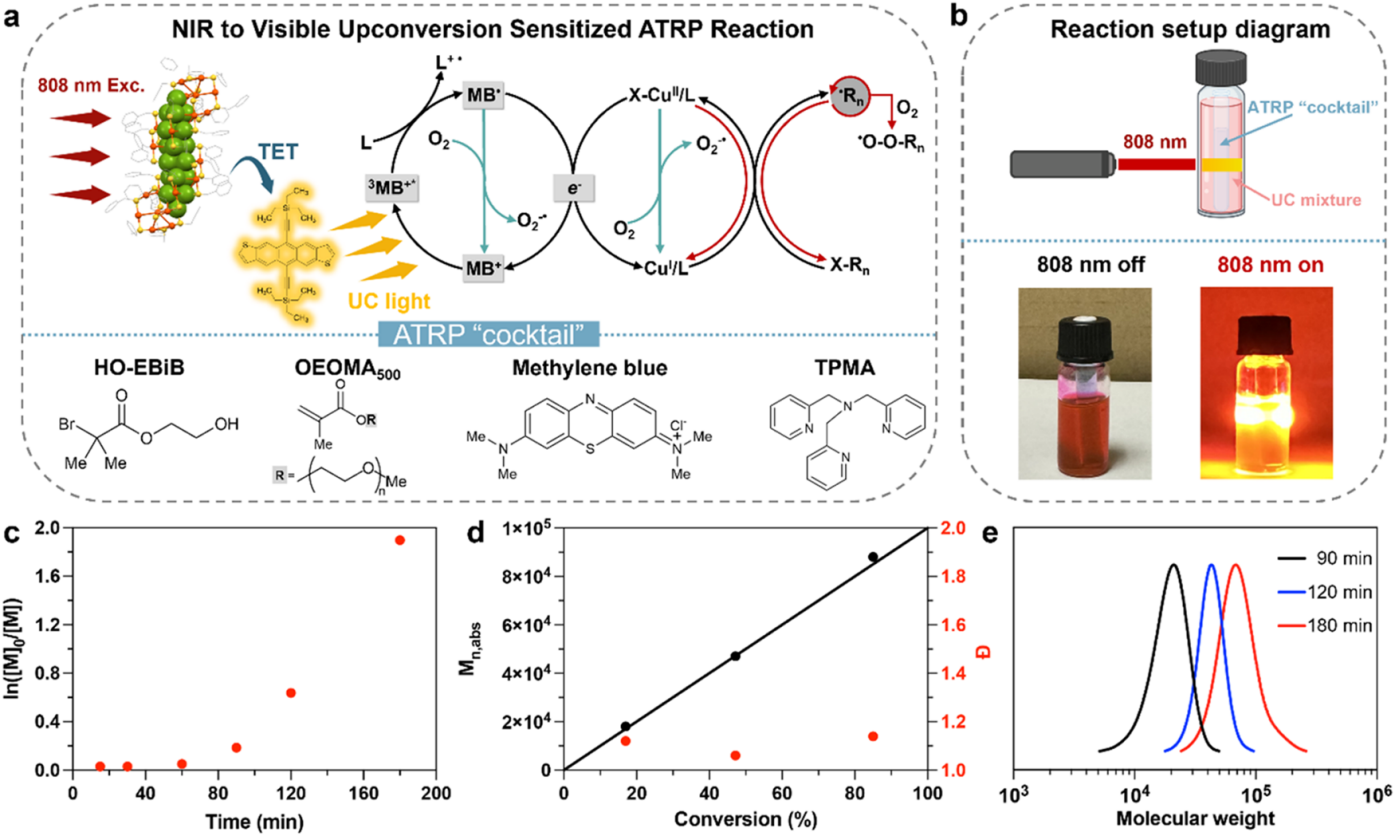
(a) Schematic illustration of upconversion photon induced MB^+^/Cu-catalyzed photo-ATRP. (b) Top panel: diagram of the reaction setup for the Au_42_/TES-ADT TTA-UC system triggering the ATRP reaction. Lower panel: photograph of the actual reaction setup with the 808 nm laser switched on and off. (c) First-order kinetic plot of MB^+^/Cu-catalyzed photo-ATRP (d) Evolution of molecular weight and molecular weight distribution with monomer conversion, and (e) SEC traces evolution with time. Reaction conditions: [OEOMA_500_]/[HO-EBiB]/[MB^+^]/[CuBr_2_]/[TPMA] = 100/1/0.017/0.2/0.6, [OEOMA_500_] = 300 mM, in 1× PBS with DMSO (1.3% v/v), irradiated under a 1 W/cm^2^ 808 nm laser.

A polymerization proceeded and, the monomer conversion reached 85% after 3 h of radiation, much faster than the previous report of using rare-earth element based upconversion nanoparticles.^54^ Kinetic analysis (Figure 4c) revealed an induction period of ∼60 min, corresponding to the time required for the catalytic system to remove oxygen from the polymerization mixture. Without the upconverted light, no polymer was formed, due to the negligible absorption of methylene blue at 808 nm. In addition, when the reaction was conducted at 50 °C without light radiation or the TTA-UC mixture, no monomer conversion was observed, indicating that the polymerization was not thermally initiated under NIR light irradiation. The absolute molecular weights (*M*_n,abs_) increased linearly with monomer conversion while maintaining a narrow molecular weight distribution (1.05 ≤ *Đ* ≤ 1.17, Figure 4c). Furthermore, *M*_n,abs_ closely matched the theoretical values (*M*_n,th_, solid line in Figure 4c). Size-exclusion chromatography (SEC) traces (Figure 4d) showed a monomodal distribution that progressively shifted toward the high molecular weight region with prolonged irradiation, further supporting the controlled polymer growth.

To simplify the reaction setup, we attempted to perform the ATRP reaction by directly mixing the Au_42_/TES-ADT TTA-UC combination with ATRP cocktail in toluene, eliminating the need for the inner reaction tube. However, we observed a rapid decline in UC emission. UV–vis spectra revealed that, as the UC light diminished, the characteristic absorption peak of Au_42_ also disappeared. Adding additional Au_42_ temporarily restored the UC light, but it quickly diminished again. This instability was attributed to an unintended metal exchange reaction between the [X–Cu^II^/TPMA]^+^ complex in the ATRP cocktail and Au_42_, which decomposed the Au_42_. Notably, this reaction occurred even in the absence of light exposure, indicating that it was chemically driven rather than photochemically induced.

### Au_42_/TES-ADT@SiO_2_ Upconversion NPs for Aqueous ATRP

To resolve the compatibility issue between the Au_42_/TES-ADT TTA-UC sy*s*tem and the ATRP cocktail, we sought to encapsulate the nanodroplets of the upconversion stock solutions to physically separate Au_42_ from the copper catalyst, preventing undesirable side reactions. We were inspired by the recent work of the Congreve group,^4^ who developed a nanocapsule synthesis method that stably encapsulates TTA-UC nanodroplets with a silica shell. We adopted their concept but designed a protocol that suits our system.

As shown in Figure 5a, Au_42_ and TES-ADT were first dissolved in 6-phenylhexanoic acid to form a TTA-UC stock solution. Interestingly, the stock solution exhibited TTA emission even under aerated conditions, due to the very limited solubility of oxygen in the fatty acid. The mixture was then combined with water and subjected to vigorous blending and ultrasonication to form an emulsion. Next, (3-aminopropyl)­triethoxysilane (APTES) was added, and the mixture became transparent, indicating the encapsulation of the micelles by APTES. Finally, anhydrous tetraethyl orthosilicate (TEOS) and MPEG-silane (molecular weight: 10 K) were added sequentially to form a complete SiO_2_ protective shell. Full details of the optimized nanocapsule synthesis are provided in the Supporting Information.

**Figure 5:**
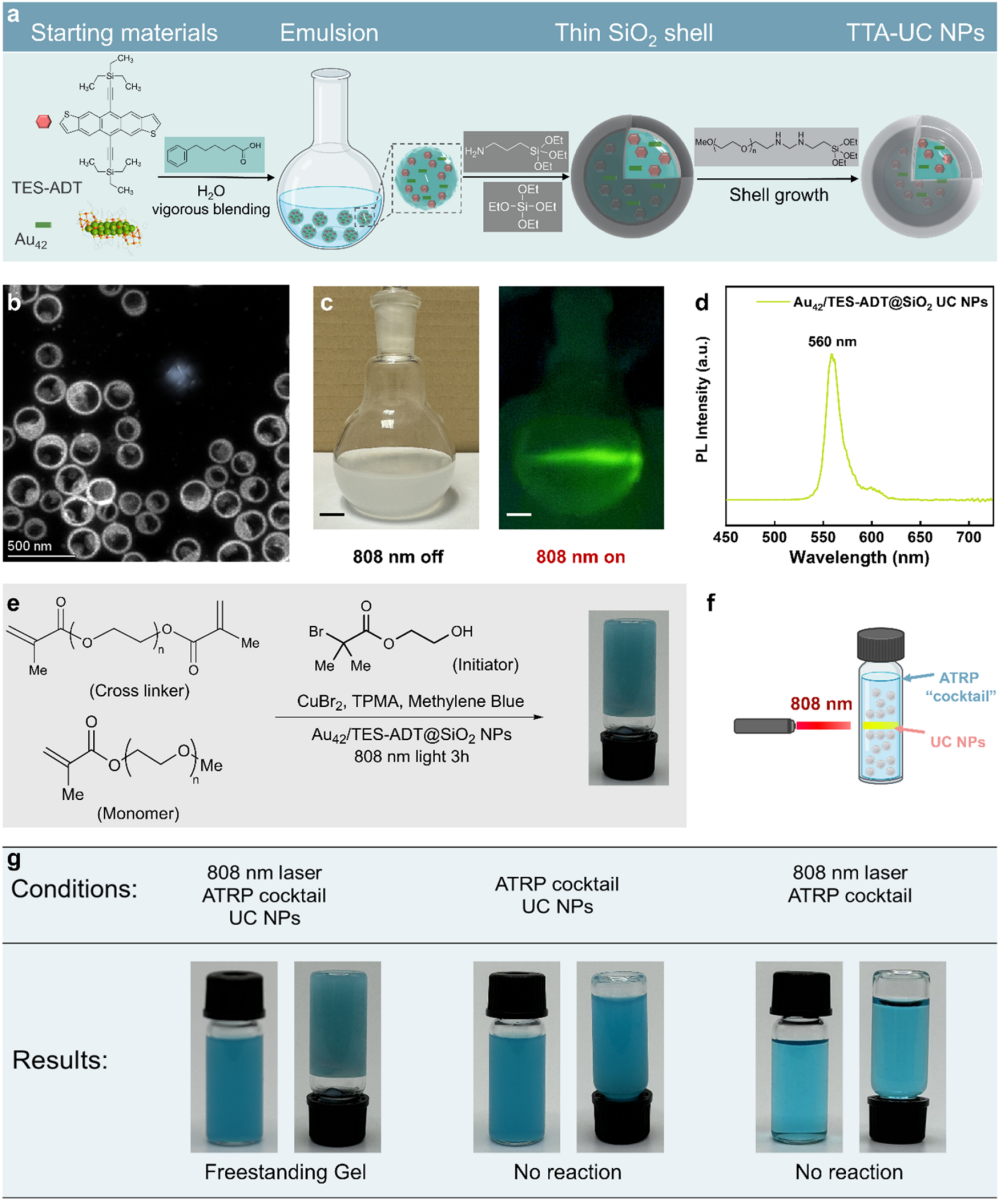
(a) Overview of the synthesis of Au_42_/TES-ADT TTA-UC NPs. 6-Phenylhexanoic acid nanodroplet micelles containing Au_42_/TES-ADT upconversion materials are encapsulated in silica and further decorated with covalently bound PEG chains to facilitate water suspension. (b) Scanning transmission electron microscopy (STEM) image of Au_42_/TES-ADT@SiO_2_ NPs. (c) Photograph of a suspension of Au_42_/TES-ADT@SiO_2_ NPs under ambient light (left panel) and 808 nm laser (right panel, imaged through a 450 to 720 nm bandpass filter with power intensity of 250 mW/cm^2^, scale bar: 1 cm). (d) Visible region emission spectra of Au_42_/TES-ADT@SiO_2_ TTA-UC NPs under 808 nm continuous laser excitation. (e) Schematic illustration of Au_42_/TES-ADT@SiO_2_ NPs enabled photo-ATRP reaction to form a cross-linked hydrogel. Reaction conditions: [OEOMA_500_]/[PEGDMA_750_]/[HO-EBiB]/[MB^+^]/[CuBr_2_]/[TPMA] = 200/40/1/0.025/0.3/0.9, [OEOMA_500_] = 300 mM, [PEGDMA_750_] = 60 mM, [Au_42_/TES-ADT@SiO_2_ NPs] = 5 wt %, in 1× PBS with DMSO (1.3% v/v), irradiated under a 1 W/cm^2^ 808 nm laser. (f) Schematic diagram of reaction setup. (g) Summary table of the experimental conditions and outcomes of NIR light driven photopolymerization using Au_42_/TES-ADT TTA-UC NPs upon photoexcitation at 808 nm.

Scanning transmission electron microscopy (STEM) analysis of the synthesized Au_42_/TES-ADT@SiO_2_ TTA-UC nanoparticles (NPs) revealed an average diameter (∼250 nm) of the capsules (Figures 5b and S7). However, elemental analysis (Figure S8) detected only Si and O signals, with no detectable Au signal (likely due to the low loading of Au_42_). The resulting Au_42_/TES-ADT@SiO_2_ NPs exhibited excellent dispersibility in water (Figure 5c, left panel) and the suspension stayed well dispersed for 5 days (longer time not investigated). When excited with an 808 nm laser, the suspension emitted a bright yellowish-green light (Figure 5c, right panel), observable through a 450–720 nm bandpass filter.

Upon 808 nm excitation, the visible-region PL spectrum (Figure 5d) of the upconverted emission centered at 560 nm, corresponding to a 0.68 eV anti-Stokes shift. The emission profile closely matched that of TES-ADT at low concentrations (Figure 1f), suggesting that while most TES-ADT molecules were not encapsulated within the silica shell, the remaining fraction was sufficient to achieve efficient TTA-UC. This stands in contrast to solution systems, which typically require extremely high dye concentrations for effective upconversion. The Φ’_UC_ of the Au_42_/TES-ADT@SiO_2_ NPs was estimated to be 5.1% by a relative method, using the solution data (Figure 2b) as the reference, suggesting that the TTA-UC efficiency was preserved. It is worth noting that light scattering from the NPs strongly attenuated the measured emission intensity, implying that the actual number of upconverted photons generated was much higher than detected.

The ability of the Au_42_/TES-ADT@SiO_2_ NPs to achieve efficient TTA-UC with a relatively low TES-ADT concentration is likely due to a confinement effect imposed by the SiO_2_ shell. This confinement limits the distance between the sensitizers and annihilator molecules, increasing their collision probability and thereby reducing the amount of annihilator required. Additionally, since the PLQY of TES-ADT in the solid state is below 3%,^55^ it is unlikely that the observed emission originates from TES-ADT and Au_42_ embedded within the silica shell. Instead, it primarily arises from the encapsulated nanodroplets. Additionally, the NIR-region PL spectrum (Figure S9) upon 808 nm excitation revealed emission from Au_42_. However, a shift in the fluorescence-to-phosphorescence peak ratio was observed, suggesting strong dipole–dipole interactions between adjacent Au_42_ clusters.^28^ Such interactions are known to enhance the intersystem crossing (ISC) rate, thereby increasing Φ_ISC_ and contributing to the high TTA-UC efficiency in the Au_42_/TES-ADT@SiO_2_ NPs.

To test the efficiency of the TTA-UC Au_42_/TES-ADT@SiO_2_ NPs for photo-ATRP reaction, we mixed the NPs with the previous ATRP cocktail and added poly­(ethylene glycol) dimethacrylate (average *M*_n_ = 750, PEGDMA750) as the crossing-linking reagent (Figure 5e). The concentrations of all other reactants were the same as the previous case but scaled up to a 2 mL vial. 100 mg TTA-UC NPs (5 wt %) were added to the mixture to provide the upconverted photons. The setup for the polymerization reactions is shown in Figure 5f, in which the illumination source was an 808 nm laser beam with an area of ∼0.7 cm^2^ at the reactor. With the SiO_2_ shell, the TTA-UC system represented excellent compatibility with the ATRP cocktail, ensuring that the upconversion process remains efficient and stable in the polymerization environment. Notably, the upconversion reaction proceeded effectively even in the presence of oxygen, overcoming a major limitation commonly associated with triplet-based upconversion systems, which typically require an oxygen-free environment.^56^ After 3 h of laser illumination, a polymer gel was observed, indicating the successful initiation and progression of the ATRP reaction. In contrast, control experiments conducted in the dark or without the Au_42_/TES-ADT@SiO_2_ NPs resulted in no gel formation (Figure 5g), confirming that both light irradiation and the presence of the TTA-UC NPs were essential for triggering the polymerization process. These findings highlight the unique advantages of the Au_42_/TES-ADT@SiO_2_ NPs as the TTA-UC system, paving the way for aqueous phase NIR light-triggered photopolymerization and expanding its potential applications in biomedicine, coatings, and 3D printing technologies.

## Conclusions

In this work, we have developed a highly efficient Au_42_/TES-ADT TTA-UC system capable of upconverting near-infrared to visible photons with low threshold energy. The system demonstrated excellent photostability, and the upconverted photons were successfully utilized to trigger an efficient and highly controllable ATRP reaction. Furthermore, SiO_2_ encapsulation significantly enhanced the system’s compatibility with reactive substances, enabling aqueous NIR light triggered photo-ATRP to proceed in the presence of oxygen. The excellent biocompatibility of the Au_42_/TES-ADT@SiO_2_ TTA-UC NPs and good penetration capability of NIR light allowed the reaction to proceed even in highly scattering media, such as 3D-printed objects or within biological tissues.

A potential drawback of the Au_42_/TES-ADT@SiO_2_ TTA-UC NPs is light scattering caused by the silica shell, which reduces the emission intensity. A conventional approach to mitigate scattering involves synthesizing smaller SiO_2_ nanoparticles (i.e., <20 nm); however, this can be challenging and requires sophisticated techniques. A more straightforward strategy to alleviate scattering and minimize emission attenuation is to introduce strongly absorbing molecules to increase the refractive index (RI) of the aqueous medium (RI ≈ 1.33 in the visible region) to better match that of SiO_2_ (RI ≈ 1.43). Previous studies have demonstrated that tartrazine effectively enhances the RI of aqueous solutions.^57^ However, its absorption spectrum overlaps with the emission of TES-ADT, leading to undesired reabsorption. Alternatively, the same RI-matching effect can be achieved by employing strong UV absorbing molecules, thereby avoiding reabsorption of the emitted light.

Overall, the gold quantum rod-based TTA-UC system demonstrates high efficiency, excellent stability, and outstanding biocompatibility. As previously reported, other gold quantum rods such as Au_60_, Au_78_, Au_96_, and Au_114_ also exhibit exceptional photosensitizing capabilities in the short-wave infrared region.^26^ Future studies may explore the potential of these quantum rods for TTA upconversion, with the aim of further improving energy conversion efficiency. Moreover, chemically anchoring dye molecules directly onto the quantum rod surface could significantly enhance photon upconversion performance.^58^

## Supplementary Material



## Notes

Experimental section; absorption and photoluminescence spectra; nanosecond transient absorption (TA) data map; photostability test of Au_42_/TES-ADT TTA-UC system; NIR region emission spectra of Au_42_/TES-ADT@SiO_2_ TTA-UC NPs under 808 nm excitation, and summary of reported NIR-to-visible TTA-UC efficiencies (PDF)
