# Supplementary material for: Near-Infrared to Visible Photon Upconversion with Gold Quantum Rods and Aqueous Photo-Driven Polymerization
Source: J Am Chem Soc. 2025 Jul 28;147(31):28241–50. doi: 10.1021/jacs.5c08826 (PMC12333327; doi:10.1021/jacs.5c08826)
Supplement: Supplementary file 1 [file ja5c08826_si_001.pdf]

Near-Infrared to Visible Photon Upconversion with Gold Quantum Rods and Aqueous Photo-Driven Polymerization

Zhongyu Liu[1$], Xiaolei Hu[1$], Lianshun Luo[1$], Guiying He[1], Abhrojyoti Mazumder[1], Ece Gunay[2], Yitong Wang[1], Elizabeth C. Dickey[2], Linda A. Peteanu[1], Krzysztof Matyjaszewski[1], and Rongchao Jin[1]*

[1]Department of Chemistry, Carnegie Mellon University, Pittsburgh, Pennsylvania 15213, USA

[2]Department of Materials Science and Engineering, Carnegie Mellon University, Pittsburgh, Pennsylvania 15213, USA

[$]Z. Liu, X. Hu and L. Luo contributed equally to this work

*Correspondence author, Email: rongchao@andrew.cmu.edu

Experimental section:

Chemicals: Tetrachloroauric (III) acid ($HAuCl_4 \cdot 3H_2O$, 99.999% metal basis, Aldrich), phenylethyl mercaptan (PET, >99%, Aldrich), sodium borohydride (powder, 98%, Aldrich), benzimidazole (98%, Aldrich), 2-bromopropane (99%, Aldrich), dimethyl sulfide (SMe_2, >99%, Aldrich), potassium carbonate (K_2CO_3, 99%, Aldrich), TES-ADT (99% Aldrich), violanthrone-79 (98%, Fisher Scientific), rubrene (98%, Aldrich), methylene blue (MB^+, 99%, Aldrich), copper(II) bromide ($CuBr_2$, 99.99%, Aldrich), tris(2-pyridylmethyl)amine (TPMA, 99%, AmBeed), 2-hydroxyethyl α-bromoisobutyrate (HO-EBiB, 95%, Aldrich), oligo(ethylene glycol) methyl ether methacrylate (average M_n= 500, $OEOMA_{500}$, Aldrich), poly(ethylene glycol) dimethacrylate (average M_n=750, $PEGDMA_{750}$ Aldrich), 6-phenylhexanoic acid (95%, Fisher Scientific), anhydrous tetraethyl orthosilicate (TEOS, 99%, Aldrich), (3-aminopropyl)triethoxysilane (APTES, 99%, Aldrich), 10K MPEG-Silane (Nanosoft Polymers). Methanol, ethanol, ethyl ether, dichloromethane (DCM), toluene, acetone, 10X phosphate-buffered saline (10X PBS), n-hexane and acetonitrile (HPLC grade for all solvents) were used as received. Thin-layer chromatography (TLC) plates were purchased from iChromatography (silica gel, 250 μm).

Synthesis of chloro(dimethylsulfide)gold(I) ($AuCl(SMe_2)$)

500 mg $HAuCl_4 \cdot 3H_2O$ was dissolved in ethanol (20 mL), followed by the addition of 280 μL SMe_2 with vigorous stirring for 2 hours. The resulting white precipitate was collected by centrifugation, washed with ethyl ether, and dried to give rise to the product as a white powder.

Synthesis of 1,3-diisopropylbenzimidazolium bromide ($^iPr_2$-bimy·HBr)

Benzimidazole (1.18 g) and K_2CO_3 (760 mg) were added to acetonitrile (8 mL) and stirred rapidly under ambient temperature for 1 hour. Subsequently, 2-bromopropane (5.4 mL) was added to the suspension, and the reaction mixture was refluxed with vigorous stirring for 24 hours. A second portion of 2-bromopropane (5.4 mL) was then added, and the mixture was refluxed under the same conditions for an additional 48 hours. After removing the solvent under reduced pressure, the residue was dissolved by DCM, and the upper supernatant was collected following centrifugation. The solvent was then evaporated under reduced pressure, yielding a spongy solid, which was washed with ethyl acetate. The final product was a white powder.

Synthesis of NHC-Au-Br complex ($^iPr_2$-bimy·AuBr)

$^{i}Pr_2$-bimy·HBr (1337.4 mg), AuCl(SMe_2) (1393.4 mg), and K_2CO_3 (653.5 mg) were added into acetone (20 mL) and vigorously stirred under reflux conditions for 2 hours. Afterwards, the solvent was removed under reduced pressure. The residue was then treated with DCM, and the upper supernatant was collected following centrifugation. The solvent was evaporated under reduced pressure, yielding a solid product, which was washed with pentane and dried to afford the desired product as gray powder.

Synthesis of $Au_{42}(PET)_{32}$ quantum rod

iPr_2-bimy·AuBr (120 mg) and PET (67 μL) were dissolved in a mixture of chloroform (15 mL) and ethanol (5 mL). Within 10 minutes of stirring, the solution gradually turned cloudy white, indicating the formation of Au(I)-PET. The suspension was then reduced by adding $(CH_3)_3CNH_2{\cdot}BH_3$ (434 mg). The reaction proceeded for 6 hours before the solvent was removed under reduced pressure. The resulting mixture was thoroughly washed with methanol, extracted with DCM, and concentrated for TLC separation. The mixture was transferred to a TLC plate and separated in a developing tank using a 1:1 (v/v) DCM:*n*-hexane solvent system. The green band corresponding to $Au_{42}(PET)_{32}$ was cut and re-dissolved in DCM.

Preparation of Samples for TTA-UC Measurements

A toluene solution containing the Au_{42} sensitizer (3 μM) and TES-ADT (10 mM) annihilator/emittor was prepared under ambient conditions. The solution was then transferred to a 1 cm quartz cuvette and thoroughly degassed with helium. Notably, the absorption spectrum of the mixture remained unchanged compared to their individual spectra, indicating the absence of ground-state complex formation between the nanocluster and the annihilator/emitter in solution.

Au_{42}/TES-ADT for photo-ATRP

First, stock solutions of $OEOMA_{500}$ (600 mM in H_2O), MB^+ (1.875 mM in H_2O), HO-EBiB (75 mM in H_2O), $CuBr_2$ (11.25 mM in H_2O), and TPMA (67.5 mM in DMSO) were prepared. A typical ATRP "cocktail" solution (250 μL) was then prepared as follows. $OEOMA_{500}$ stock (125 μL), MB^+ stock (5 μL), $CuBr_2$ stock (10 μL), TPMA stock (5 μL), HO-EBiB stock (5 μL), DMSO (15 μL), H_2O (60 μL) and 10X PBS solution (25 μL) were then mixed. The final concentrations were $OEOMA_{500}$ (300 mM), MB^+ (37.5 μM), $CuBr_2$ (0.45 mM), TPMA (1.35 mM), HO-EBiB (1.5 mM), DMSO (10% *v*/*v*), and 1X PBS. The ATRP "cocktail" was then transferred to a glass insert (300 μL). The glass inserts containing ATRP "cocktail" solutions was put into a vial (2 mL) filled with Au_{42}/TES-ADT upconversion solution (3 μM Au_{42} sensitizer and 10 mM TES-ADT, 1 mL). The vials were irradiated under the laser (808 nm, 1 W cm^{-2}). At different time intervals (0, 15, 30, 60, 90, 120, and 160 min), samples were taken for ^1H NMR and SEC characterization.

Synthesis of Au_{42}/TES-ADT@SiO_2 TTA-UC NPs

A solution of Au_{42} (80 μM) was prepared by dissolving it in 80 μL of 6-phenylhexanoic acid under ultrasonication. Separately, TES-ADT (20 mM) was dissolved in 80 μL of 6-phenylhexanoic acid. The two solutions were combined and ultrasonicated to ensure homogeneous dispersion. The resulting mixture was kept in the dark for 30 minutes prior to further processing. Subsequently, the mixture was added to 20 mL of water and emulsified using a high-intensity ultrasonic processor. The resulting nanoemulsion was transferred into a nitrogen-saturated flask and immediately stirred at 1200 rpm with a magnetic stir bar. (3-Aminopropyl)triethoxysilane (75 μL) was added until the solution became transparent, followed by the immediate addition of 10 kDa MPEG-silane (250 mg) to prevent capsule aggregation. The dispersion was stirred vigorously to ensure uniform distribution. After 5 minutes, 3.0 mL of anhydrous tetraethyl orthosilicate (TEOS) was added in a single portion. The reaction mixture was maintained at 45 °C under vigorous stirring (500 rpm) for 36 hours. A second portion of MPEG-silane (400 mg) was introduced after approximately 36 hours. Upon completion of the reaction (~48 hours), the crude product was cooled to room temperature under a constant nitrogen atmosphere. The reaction mixture was then transferred to centrifuge tubes and centrifuged at 5000 × g for 30 minutes, after which the pellet was discarded. The remaining suspension was dried by evaporating water under a nitrogen stream.

Photo-ATRP catalyzed by Au_{42}/TES-ADT@SiO_2 TTA-UC NPs for hydrogel synthesis in aqueous medium

Based on the general procedure for photo-ATRP, ATRP "cocktail" solution (2 mL) was prepared by mixing $OEOMA_{500}$ stock (1 mL), $PEGDMA_{750}$ (81 µL), Au_{42}/TES-ADT@SiO_2 stock (100 mg in 729 µL), MB^+ stock (40 µL), $CuBr_2$ stock (80 µL), TPMA stock (40 µL), HO-EBiB stock (30 µL). The final concentrations were $OEOMA_{500}$ (300 mM), $PEGDMA_{750}$ (60 mM), MB^+ (37.5 µM), $CuBr_2$ (0.45 mM), TPMA (1.35 mM), and HO-EBiB (1.5 mM). The ATRP "cocktail" was transferred to a glass vial (2 mL) and was irradiated under the laser (808 nm, 1 W cm^{-2}) for 3 hours.

Characterization

UV-Vis-NIR spectra of the nanoclusters were collected with a UV-3600 Plus UV-VIS-NIR spectrophotometer (Shimadzu, detection range: 185 to 3300 nm). Steady state photoluminescence spectra were measured on a FLS-1000 spectrofluorometer (Edinburgh). Near infrared PL was measured using a wide range InGaAs detector cooled by liquid nitrogen (-80 °C). The time-resolved PL measurements were carried out using a multi-channel scaling (MCS) single photon counting technique. An EPL-450 picosecond pulsed diode laser (Edinburgh Instruments) was used as the light source. The excitation wavelength from EPL-450 centered at 450 nm with a variance smaller than 5 nm. The pulse duration is less than 100 ps. For all MCS measurements, the detection wavelengths were set at the center of peaks.

Scanning transmission electron microscopy (STEM) was performed on drop-cast samples using a ThermoFisher Titan-Themis TEM operated at 80 kV. High-angle annular dark-field (HAADF)-STEM images were carried out with a probe convergence angle of 30 mrads and a camera length yielding a collection angle range of 46-200 mrad. Energy-dispersive spectroscopy was conducted in STEM mode (STEM-EDS) using a Thermo Fisher SuperXG2 detector, with a beam current of 60 pA and a pixel size of 260 pm.

Transient absorption measurements were carried out using a broadband pump–probe setup, which is pumped by a commercial Ti:Sapphire laser system (Coherent Astrella, 1 kHz) with an optical parametric amplifier. TA spectra were recorded over time up to 100 µs with a resonance pump of S_1 state of Au_{42} at 808 nm and the probe pulse covering between 760 nm and 1600 nm. The probe light is generated with a fiber laser (Leukos) and the delay time is controlled by an electronic delay configuration. A solution of ~3 µM Au_{42} in toluene was used as the control solution. The sensitization experiments were performed by adding TES-ADT to the Au_{42} solution (TES-ADT concentration is 3 mM and 10 mM). The photoexcited triplet state of Au_{42} decays fast by collisional triplet energy transfer from Au_{42} to TES-ADT.

PL Quantum Yield Measurements

The relative quantum yield (Φ_S) of the sample was calculated using:

$$\Phi_S = \Phi_R(\frac{I_S}{I_R})(\frac{1-10^{-A_R}}{1-10^{-A_S}})(\frac{n_S}{n_R})^2$$

where, Φ_R is the quantum yield of the reference standard, *I* is the integrated PL intensity, *A* is the absorbance of the solution at the excitation wavelength, and *n* is the refractive index of the solution solvent. (The subscripts *S* and *R* represent sample and reference, respectively.)

Table S1. Summary of reported NIR-to-visible TTA-UC efficiencies.

Sensitizer*	Emitter	λ_{ex} (nm)	Δ_{as} (eV)	Φ'_{UC} (%)	I_{th}(mW/cm^2)
**Au_{42} (This work)**	TES-ADT	808	0.50	6.7	90
$PtAg_{24}$[1]	TIPS-Ac	785	1.03	2.4	1100
$Au_{25-x}Cu_x$ rod[2]	BPEA	805	0.89	4.6	5000
Au_{25} rod[2]	BPEA	805	0.89	1.0	4000
PbS (solid state)[3]	Rubrene:DBP	808	0.49	1.2	12000
PbS-2.73/Th-DPP[4]	Rubrene	808	0.70	13.5	4800
PbS-3.11/Th-DPP[4]	Rubrene	980	0.97	0.45	19900
PbS-3.11/Th-DPP[4]	Rubrene	1064	1.07	0.37	23500
PbS[5]	TES-ADT	1064	0.86	0.09	43000
ZClSe/TCA[6]	Rubrene	808	0.68	16.7	2100
Pyr_1RuPZn_2 [7]	PDI	780	0.70	0.75	22
PtNac[8]	TDI	856	0.35	0.89	>30000
Os complex[9]	Rubrene	938	0.85	3.1	10000
$Os(peptpy)_2^{2+}$[10]	TTBP	724	0.85	5.9	13000
PbS-TTCA[11]	V79	808	0.23	0.06	>1000
ITIC-Cl[12]	Rubrene:DBP	750	0.38	4.6	39
TTM-TPA[13]	Rubrene	733	0.93	13.6	1000
InAs/ZnSe[14]	Rubrene	808	0.68	21.1	20200
PdTNP[15]	Py5	720	0.45	14.1	40.7

(* refs 1-15 are listed in the final page)

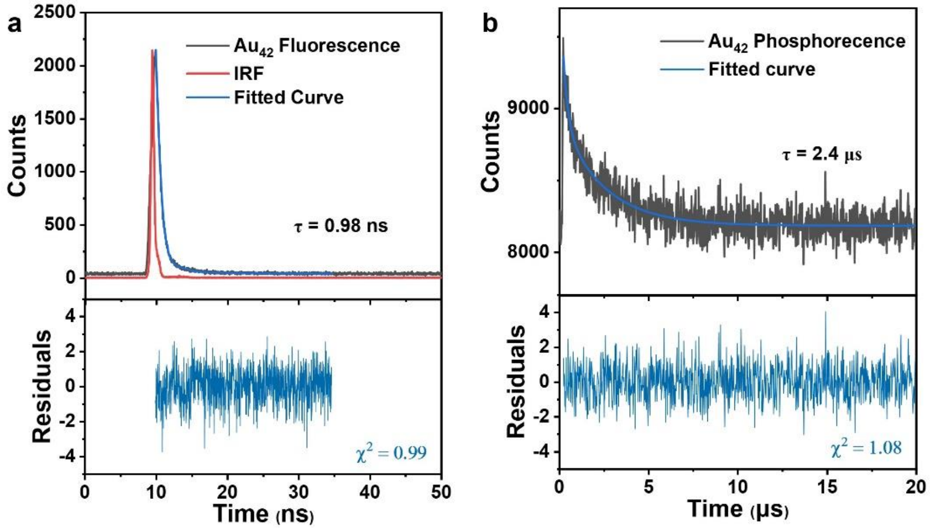

Figure S1. (a) Decay profile of fluorescence (FL), fitting and residuals. (b) Decay profile of phosphorescence (PH), fitting and residuals. Ex: ~100 ps pulsed laser of 400 nm.

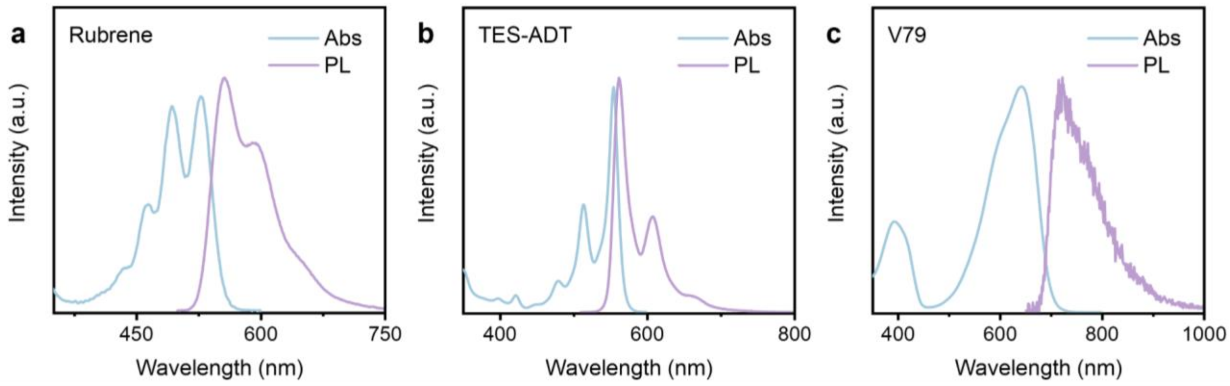

Figure S2. Absorption and photoluminescence spectra of (a) rubrene, (b) TES-ADT, and (c) V79.

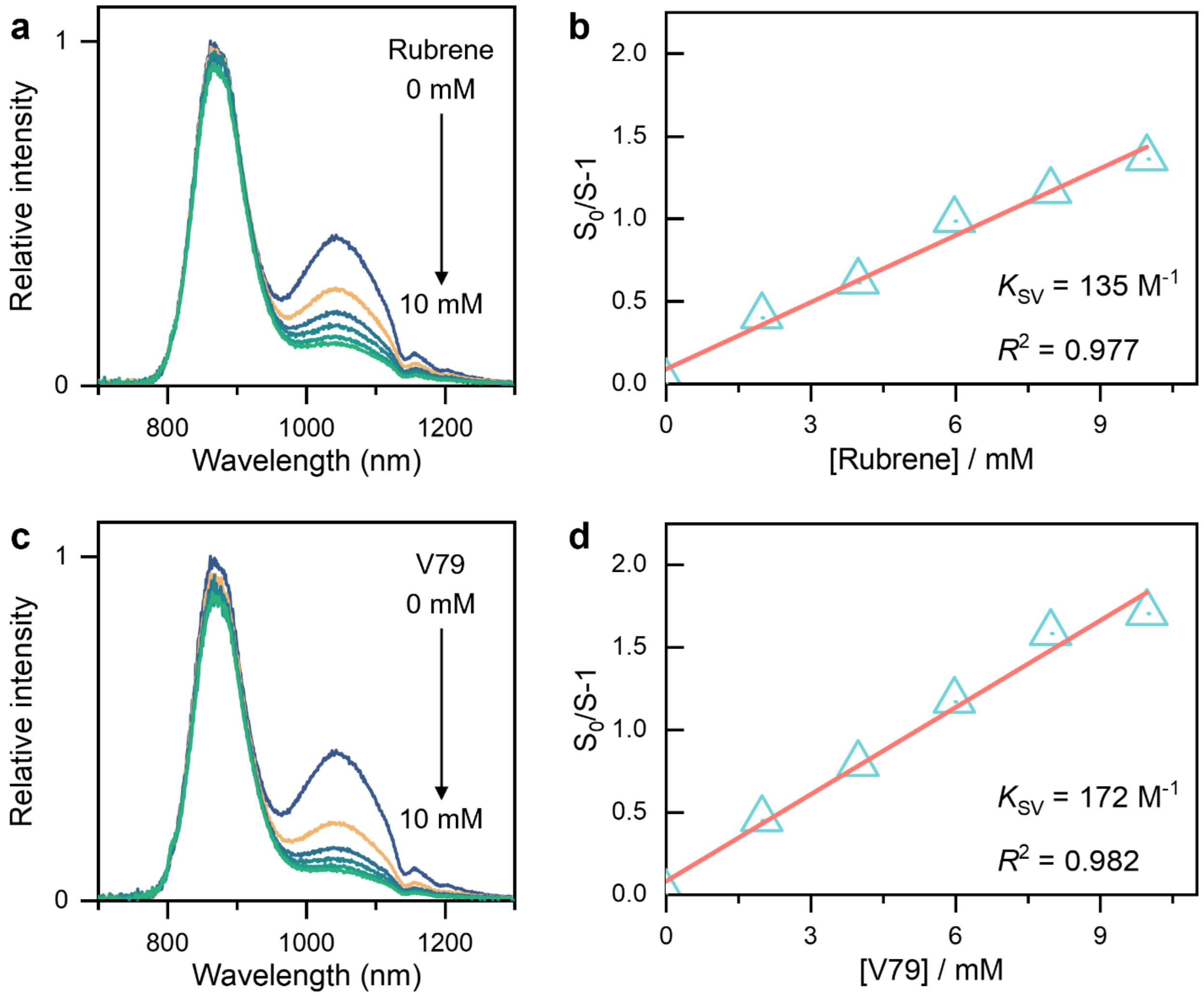

Figure S3. (a) Steady-state PL spectra of Au_{42} in deaerated toluene at varying rubrene concentrations under 808 nm excitation. (b) Stern-Volmer plot of Au_{42} PL quenching by rubrene. **(**c) Steady-state PL spectra of Au_{42} in deaerated toluene at varying V79 concentrations under 808 nm excitation. (d) Stern-Volmer plot of Au_{42} PL quenching by V79.

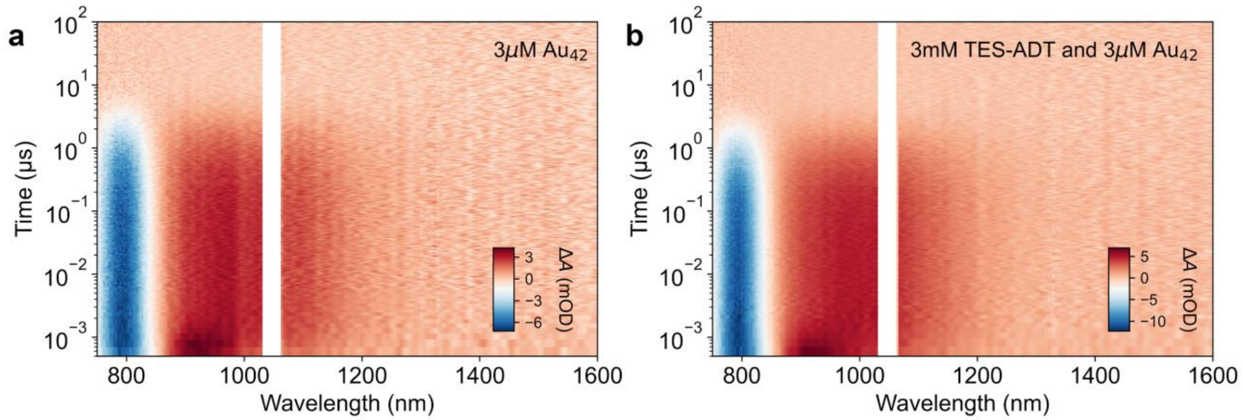

Figure S4. Nanosecond transient absorption (TA) data map of (a) 3 μM Au_{42}, and (b) 3 μM Au_{42} with 10 mM TES-ADT in an N_2-saturated toluene solution. Both solutions were under 808 nm excitation. The laser artifact at ~1030 nm in the supercontinuum probe is cut out in the data maps.

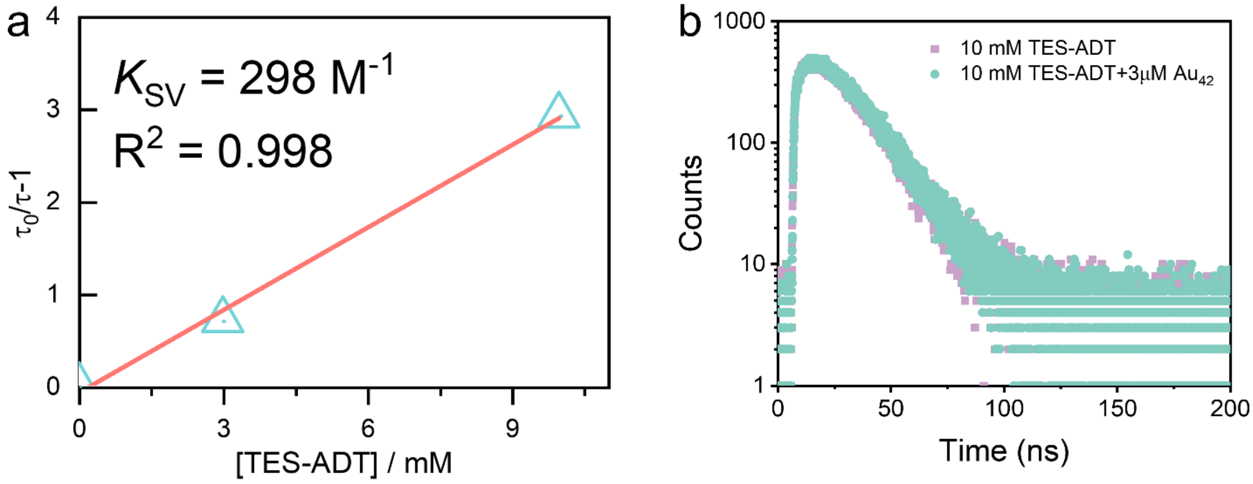

Figure S5. (a) Stern-Volmer plot of Au_{42} excited state lifetime quenching by TES-ADT. (b) Decay curves of TES-ADT before and after adding Au_{42}.

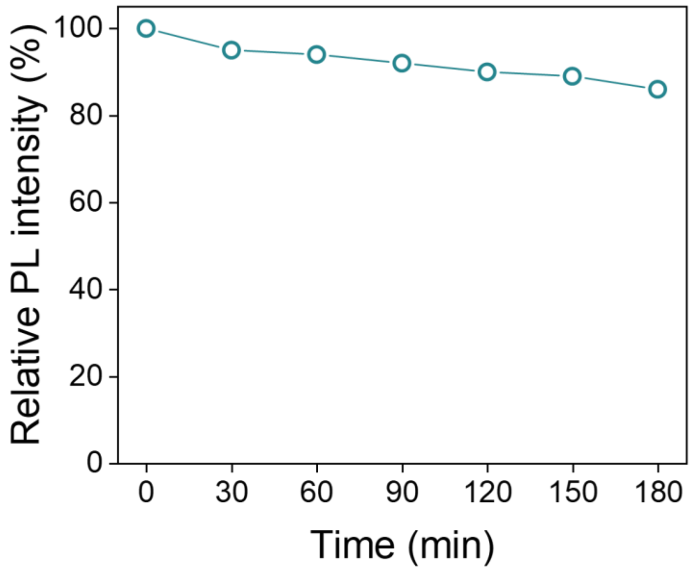

Figure S6. Photostability test of Au_{42}/TES-ADT TTA-UC system.

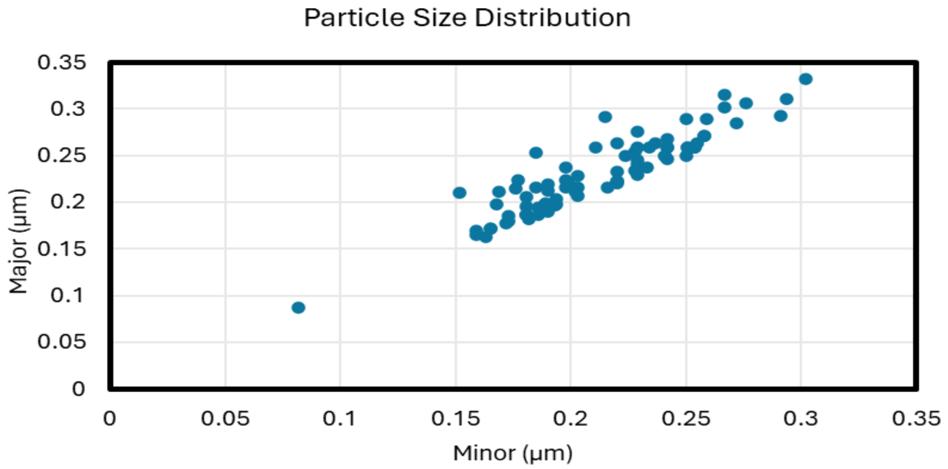

Figure S7. Particle size distribution of the Au_{42}/TES-ADT@SiO_2 TTA-UC NPs.

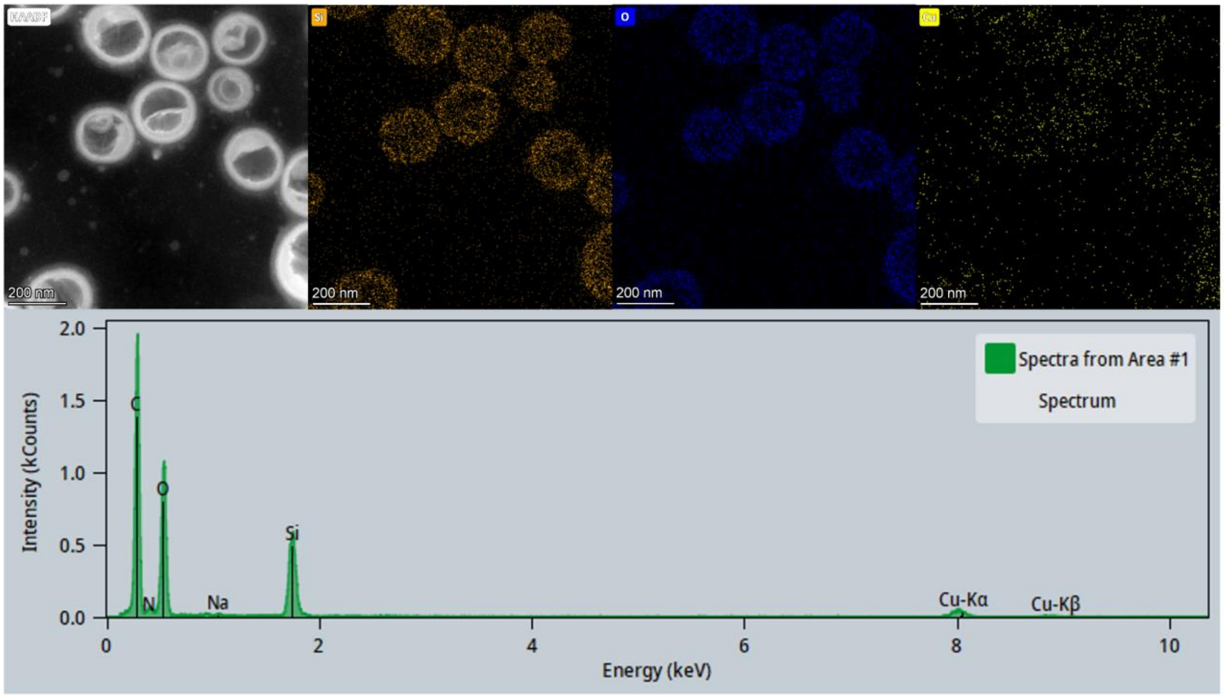

Figure S8. STEM-EDS characterization of Au_{42}/TES-ADT@SiO_2 TTA-UC NPs, where the Cu X-ray signals (Cu-Kα, Cu-Kβ and Cu-L) come from the TEM grid. Inset images: HAADF-STEM image (left), and EDS mapping analysis of Si, O and Cu on the same area of the STEM image.

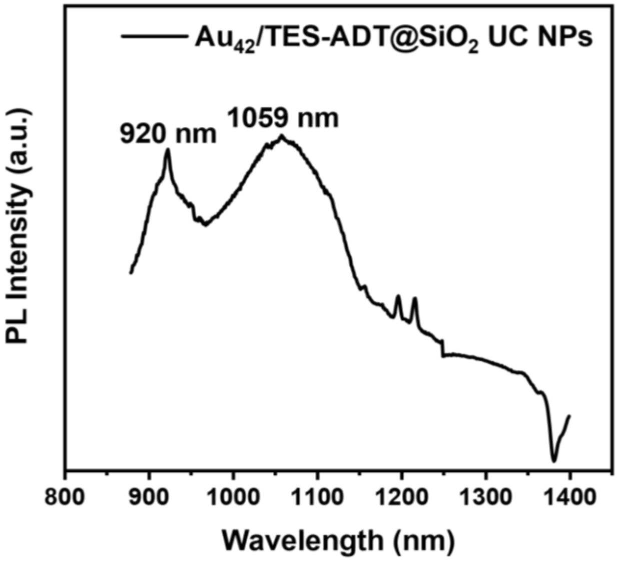

Figure S9. NIR region emission spectrum of Au_{42}/TES-ADT@SiO_2 TTA-UC NPs under 808 nm continuous-wave laser excitation.

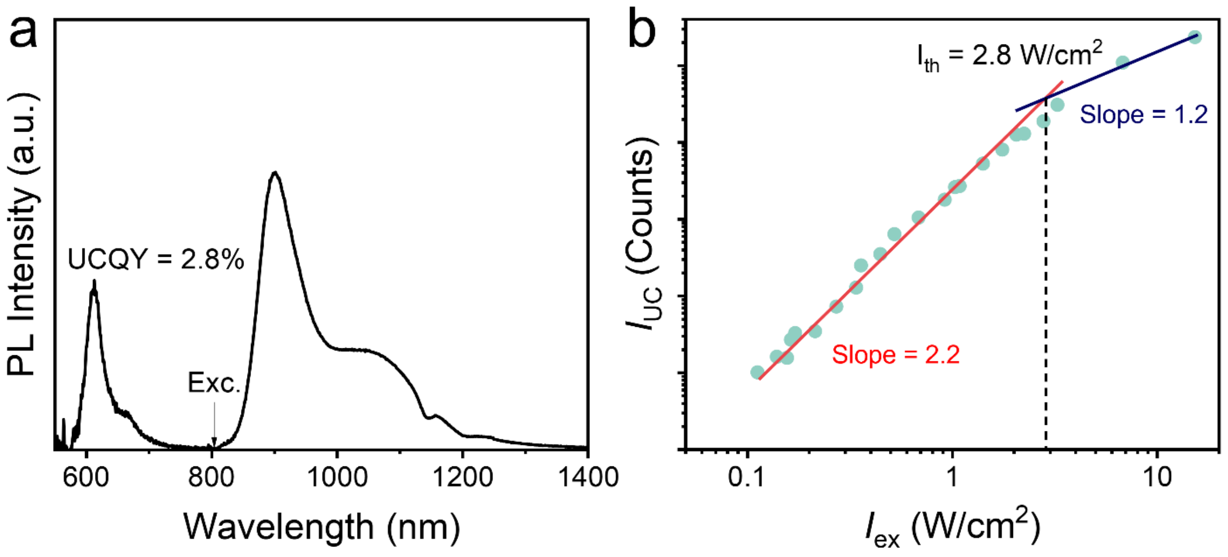

Figure S10. (a) UCQY and (b) I_{th} of stock solution for preparing Au_{42}/TES-ADT@SiO_2 TTA-UC NPs.

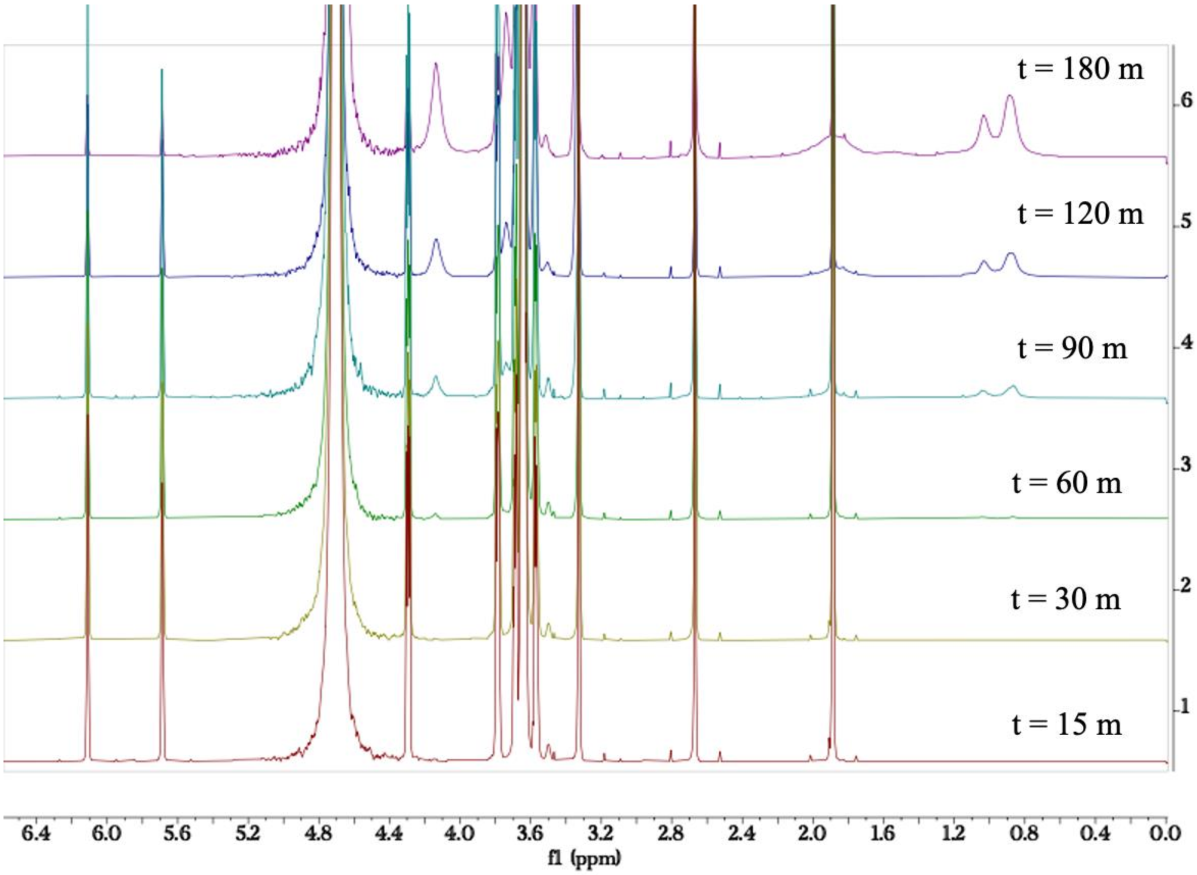

Figure S11. ^{1}H NMR for the samples taken at varying polymerization times during the kinetic study in Fig.4.

References:

1. Niihori, Y., Wada Y.&Mitsui M. Single platinum atom doping to silver clusters enables near-infrared-to-blue photon upconversion. *Angew Chem Int Ed* **60**, 2822-2827 (2021).
2. Mitsui, M., Miyoshi Y.&Arima D. Tailoring sensitization properties and improving near-infrared photon upconversion performance through alloying in superatomic molecular Au_{25} nanoclusters. *Nanoscale* **16**, 14757-14765 (2024).
3. Wu, M. et al. Solid-state infrared-to-visible upconversion sensitized by colloidal nanocrystals. *Nat Photonics* **10**, 31-34 (2016).
4. Jiang, L.-H. et al. Near infrared-II excited triplet fusion upconversion with anti-Stokes shift approaching the theoretical limit. *J Am Chem Soc* **146**, 10785-10797 (2024).
5. Nishimura, N. et al. Photon upconversion utilizing energy beyond the band gap of crystalline silicon with a hybrid TES-ADT/PbS quantum dots system. *Chem Sci* **10**, 4750-4760 (2019).
6. Liang, W. et al. Near-infrared photon upconversion and solar synthesis using lead-free nanocrystals. *Nat Photonics* **17**, 346-353 (2023).
7. Singh-Rachford, T. N. et al. Supermolecular-chromophore-sensitized near-infrared-to-visible photon upconversion. *J Am Chem Soc* **132**, 14203-14211 (2010).
8. Amemori, S., Yanai N.&Kimizuka N. Metallonaphthalocyanines as triplet sensitizers for near-infrared photon upconversion beyond 850 nm. *Phys Chem Chem Phys* **17**, 22557-22560 (2015).
9. Amemori, S., Sasaki Y., Yanai N.&Kimizuka N. Near-infrared-to-visible photon upconversion sensitized by a metal complex with spin-forbidden yet strong S_0–T_1 absorption. *J Am Chem Soc* **138**, 8702-8705 (2016).
10. Sasaki, Y. et al. Near-infrared optogenetic genome engineering based on photon-upconversion hydrogels. *Angew Chem Int Ed* **58**, 17827-17833 (2019).
11. Gholizadeh, E. M. et al. Photochemical upconversion of near-infrared light from below the silicon bandgap. *Nat Photonics* **14**, 585-590 (2020).
12. Izawa, S.&Hiramoto M. Efficient solid-state photon upconversion enabled by triplet formation at an organic semiconductor interface. *Nat Photonics* **15**, 895-900 (2021).
13. Wei, Y. et al. Π-radical photosensitizer for highly efficient and stable near-infrared photon upconversion. *Adv Opt Mater* **12**, 2301134 (2024).
14. Sun, R., Zang J., Lai R., Yang W.&Ji B. Near-infrared-to-visible photon upconversion with efficiency exceeding 21% sensitized by InAs quantum dots. *J Am Chem Soc* **146**, 17618-17623 (2024).
15. Huang, L. et al. Highly effective near-infrared activating triplet–triplet annihilation upconversion for photoredox catalysis. *J Am Chem Soc* **142**, 18460-18470 (2020).
